# Brain iron chelation by deferiprone in a phase 2 randomised double-blinded placebo controlled clinical trial in Parkinson’s disease

**DOI:** 10.1038/s41598-017-01402-2

**Published:** 2017-05-03

**Authors:** Antonio Martin-Bastida, Roberta J. Ward, Rexford Newbould, Paola Piccini, David Sharp, Christina Kabba, Maneesh C. Patel, Michael Spino, John Connelly, Fernando Tricta, Robert R. Crichton, David T. Dexter

**Affiliations:** 1 Centre for Neuroinflammation & Neurodegeneration, Division of Brain Sciences, Faculty of Medicine, Imperial College London, Hammersmith Hospital Campus, Du Cane Road, London, W12 0NN UK; 2 Imanova Ltd, Burlington Danes Building, Imperial College London, Hammersmith Hospital Campus, Du Cane Road, London, W12 0NN UK; 3 ApoPharma Inc. 200 Barmac Drive, Toronto, Ontario M9L 2Z7 Canada; 4 Universite Catholique de Louvain, 1348 Louvain-la-Neuve, Belgium; 5 Imaging Department, Charing Cross Hospital, Imperial College NHS Trust, Fulham Palace Road, London, W6 8RF UK

## Abstract

Parkinson’s disease (PD) is associated with increased iron levels in the substantia nigra (SNc). This study evaluated whether the iron chelator, deferiprone, is well tolerated, able to chelate iron from various brain regions and improve PD symptomology. In a randomised double-blind, placebo controlled trial, 22 early onset PD patients, were administered deferiprone, 10 or 15 mg/kg BID or placebo, for 6 months. Patients were evaluated for PD severity, cognitive function, depression rating and quality of life. Iron concentrations were assessed in the substantia nigra (SNc), dentate and caudate nucleus, red nucleus, putamen and globus pallidus by T2* MRI at baseline and after 3 and 6 months of treatment. Deferiprone therapy was well tolerated and was associated with a reduced dentate and caudate nucleus iron content compared to placebo. Reductions in iron content of the SNc occurred in only 3 patients, with no changes being detected in the putamen or globus pallidus. Although 30 mg/kg deferiprone treated patients showed a trend for improvement in motor-UPDRS scores and quality of life, this did not reach significance. Cognitive function and mood were not adversely affected by deferiprone therapy. Such data supports more extensive clinical trials into the potential benefits of iron chelation in PD.

## Introduction

Parkinson’s disease (PD) is the most common motor disorder, affecting approximately 1–2% of the population. Clinical diagnosis of PD is based on the presence of unilateral onset of bradykinesia, rigidity, resting tremor and postural instability. The main neuropathological hallmarks of PD are the loss of dopaminergic neurons in the substantia nigra pars compacta (SNc), the presence of intracellular Lewy bodies, deposition of α-synuclein^1^ and high iron deposition in the SNc^2, 3^. The latter has been confirmed from post mortem studies, magnetic resonance imaging (MRI), and transcranial ultrasound studies^4–6^. Additionally, electron probe x-ray microanalysis has confirmed that individual SNc dopaminergic neurons in PD have raised iron levels^7^ as well as an increased labile iron pool^8^. Such low molecular weight iron can cause oxidative damage to proteins, lipids and DNA by generating reactive oxygen species (ROS)^9^, processes for which there is evidence in the PD brain^9–11^. ROS activity will be exacerbated by decreased anti-oxidant levels, e.g. reduced glutathione, in the PD brain^12^ while α-synuclein aggregation is promoted by the excess iron^13, 14^. Therefore the removal of such iron from the SN may be beneficial in retarding the progression of PD.

Animal studies have shown that iron chelators are able to cross the blood brain barrier and are able to remove excess iron from various brain regions in models of brain iron overload^15–17^. In early studies, we demonstrated that iron chelators, desferrioxamine, deferasirox and deferiprone, currently in clinical use for treating iron overload in thalassemia major, significantly attenuated SNc dopaminergic neuronal and striatal dopamine loss in the 6-hydroxydopamine PD model, whilst reducing hydroxyl radical formation^17^. The potential benefit of using an iron chelator in a neurodegenerative condition, in the absence of systemic iron overload, was exemplified in a clinical trial utilizing deferiprone in Friedreich Ataxia (FA), where excessive concentrations of iron are present in mitochondria, caused by mutations in the frataxin gene. Cardiomyopathy, as well as neurodegeneration in the dorsal root ganglia and the dentate nucleus are prominent features in Friedreich Ataxia, and may be related to the dysregulation of iron and the resultant excess in regionalized intracellular labile iron. Deferiprone treatment, with 20 to 30 mg/kg/day, divided into 2 daily oral doses, for up to 6 months, in 9 FA patients, reduced dentate nucleus iron content, as assessed by MRI, whilst concomitantly reducing neuropathy and ataxic gait in some patients^18^.

Since iron has been implicated in the pathology of PD, its removal by iron chelators, administered at low doses, might be of therapeutic benefit in PD. The main objectives of our double-blinded, randomized, placebo-controlled, pilot clinical trial with deferiprone in PD were firstly to evaluate changes in brain iron content by T2* MRI, secondly to assess drug safety, and thirdly to assess subjects’ clinical status as characterised by PD disability utilising the Movement Disorders Society - Unified Parkinson’s Disease Rating Scale (MDS-UPDRS), mood, cognitive function and quality of life. Lastly, markers of iron homeostasis and peripheral inflammation were investigated to ascertain whether inflammation might be a contributing factor in the iron accumulation in the brain and also may hinder removal of iron from the brain. Additionally, pro-inflammatory cytokines, particularly IL-6, have been shown to play a pivotal role in peripheral and CNS iron metabolism since it stimulates the release of hepcidin, the master regulator of iron metabolism, for the liver and chorois plexus leading do decreased cellular iron export and macrophage iron retention^19, 20^.

## Patients and Methods

We prospectively studied 22 patients (12 males and 10 females; aged 50–75 years) with early stage PD, disease duration of less than 5 years (Queen Square Brain Bank criteria at Hoehn and Yahr stage 1 to 2 in the “on-medication” state) who were receiving stable PD medication regimes. Participants were excluded if they had another suspected cause for their parkinsonism, other neurological or psychiatric disorders such as history of major depression, insulin-dependent diabetes mellitus, renal or liver disease, or were immunocompromised as revealed by neutropenia, HIV, etc. Written informed consent was obtained from each patient on their initial screening visit before enrolment. The protocol was approved by Imperial College London (Reference crol1715; Date 11/05/11), the Oxford Institutional Ethical Committee (REC reference 11/SC/0101; Date 26/05/2011), MHRA (EudraCT 2011-001148-31; Date 29/12/2011) and registered at the National Health Authority. The clinical trial was registered on the publicly accessible database www.clinicaltrials.gov (NCT01539837; Date: 22/02/12). Methods were carried out in “accordance” with the approved guidelines.

**Figure 1:**
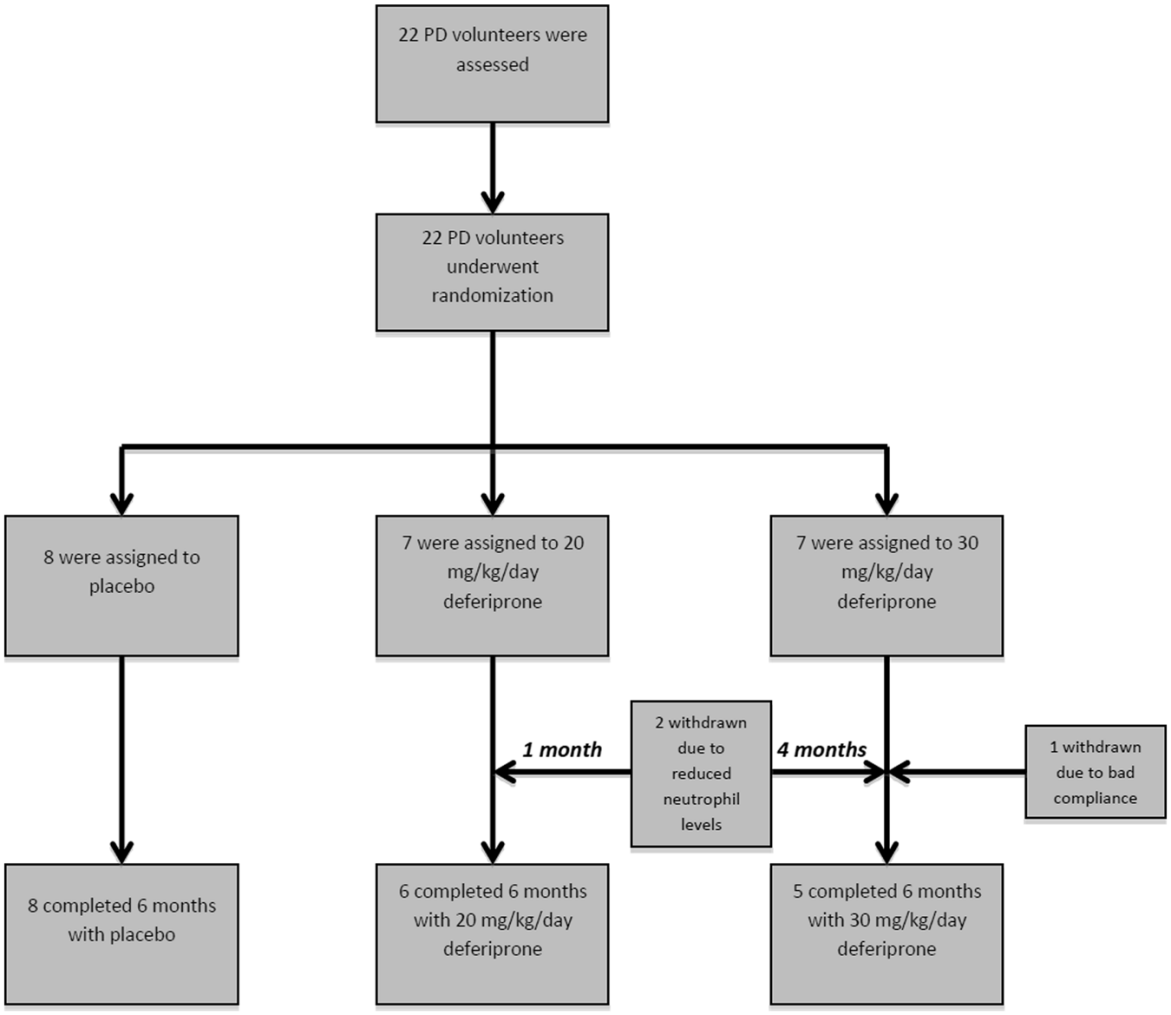
Flow diagram showing the random assignments of the two doses of deferiprone, 20 or 30 mg/kg and placebo to the 22 Parkinson’s disease patients.

The PD patients, n = 22, were recruited by AM (between 18/04/2012 – 27/03/2013) and randomly selected to receive placebo or 20 or 30 mg/kg/day deferiprone (80 mg/ml deferiprone solution or excipient matched placebo provided by ApoPharma Inc., Toronto, ON, Canada) which was divided into 2 daily oral doses, morning and evening, and administered for 6 months (Fig. 1). Randomisation was carried out by the Hammersmith Hospital Pharmacy and the randomization scheme was generated using the web site Randomization.com (http://www.randomization.com). The code for the randomisation was only disclosed at the conclusion of the trial or if the patient experienced unexpected adverse side effects. Patients, care providers and those assessing clinical outcomes were blinded to the intervention given. Of the original group of 22 patients, 19 completed the 6-month course of deferiprone, with three brain MRI scans, at 0, 3 and 6 months, repeated neurological examinations, as well as a variety of blood investigations. At this stage the trial was concluded. The analysis of the study was performed on the subjects that remained in.

The primary outcome of this double-blinded placebo-controlled study is to assess whether deferiprone is able to modify regional brain mineralization as assessed with T2* MR in PD patients. The secondary outcome is based in the drug safety of deferiprone in PD.

### Neurological measures

All PD patients were clinically assessed at the Hammersmith Hospital Campus, Imperial College London at baseline, 2 months, 4 months and 6 months for PD severity using MDS-UPDRS part 3 whilst on-medication. Anti-Parkinsonian medication dosages were maintained at a constant level for each individual throughout the study. Calculation of levodopa equivalent dosage (LED) was performed using published formulae^21^, Table 1. In addition, each subject was assessed by the Minimental State of Folstein (cognitive function), the Montgomery Asberg DRS (depression rating) and the PDQ39 (quality of life) questionnaires, Table 1.

**Table 1:** Demographic data of Parkinson’s disease patients.

	Placebo	20 mg/kg/day Deferiprone	30 mg/kg/day Deferiprone	Stats	p value
Number	8	7	7		
Gender (m:f)	3:5	4:3	5:2	X^2^ = 1.721	0.414
Age (years)	64.38 ± 3.23	68.57 ± 2.17	62.85 ± 2.74	F = 1.008	0.384
Disease duration (years)	3.54 ± 0.34	2.82 ± 0.69	3.02 ± 2.69	F = 0.675	0.521
LEDD	284 ± 55	395 ± 57	432 ± 118	F = 0.964	0.399
UPDRS III	11.38 ± 1.29	11.43 ± 1.31	10.57 ± 1.34	F = 0.129	0.879
MADRS	8.25 ± 1.78	7.14 ± 1.49	5.14 ± 1.37	F = 0.995	0.388
MMSE	29.25 ± 0.49	29.42 ± 0.29	28.57 ± 0.61	F = 0.833	0.450
PDQ 39	23 ± 4.03	31.42 ± 10.35	24.28 ± 6.29	F = 0.381	0.689

Values are represented as mean ± SEM.

### Blood and urine analysis

Blood specimens were provided by each PD patient weekly for haematological assessment to ensure that there were no adverse effects, particularly neutropenia. Agranulocytosis occurs in 1–2% of beta-thalassemia patients, who receive much higher doses of deferiprone, 75–100 mg/kg/day. Biochemical assessments were made on a monthly basis to assess various markers of iron metabolism, immunoglobulin status, hepatic and renal function, as well as levels of deferiprone and deferiprone 3-*O*-glucuronide to ensure patient compliance. Spot urine samples were collected weekly to assess iron excretion.

Inductively coupled plasma mass spectrometry (Thermo XII, Massachusetts, USA), was used for the analysis of urinary iron. Urine samples were diluted in 1% nitric acid to which the internal standard ^69^gallium had been added. A calibration curve was prepared in a similar fashion and internal quality controls were included in all analytical runs. Creatinine was analysed by a standard biochemical method. The results were expressed as nmol Fe/mmol creatinine.

### Quantification of deferiprone and deferiprone glucuronide

Deferiprone and its main metabolite, deferiprone glucuronide, were assessed in the plasma to monitor patient compliance. Perchloric acid (200 μL, 0.4 M) was added to plasma samples (200 μL) to precipitate plasma proteins. After brief vortexing, followed by 10 min incubation on ice, the samples were centrifuged (35,000× g) for 15 min at 4 °C. The supernatant (10 μL) was injected onto the HPLC column and analysed by UV detection.

### Magnetic Resonance Imaging, MRI

MRI measurements were acquired at 0, 3 and 6 months using a 3T Siemens Verio (Siemens Healthcare, Erlangen, Germany) equipped with a 32-channel phased array head coil. A 3D T1-weighted spoiled gradient echo volume of isotropic 1 × 1 × 1 mm resolution was acquired at six echo times: 6.29, 13, 21, 29, 37, and 49 ms in each TR of 56 ms. A 240 × 256 × 160 mm sagittal FOV and flip angle of 35° was used, with a parallel imaging factor of 2, requiring 10 m:14 s. A double-echo inversion recovery sequence (MP2RAGE)^22^ was also acquired with identical resolution and coverage at inversion times of 409 ms and 1100 ms in a 5 s TR with a parallel imaging factor of 2, requiring 10 m:57 s. This sequence gives two co-registered volumes: one with fluid nulled similar to the ADNI MPRAGE^23^ and one with white matter nulled for subcortical visualization. The signal decay in each voxel across the six echo acquisition was fit to the non-linear signal equation:

$${\rm{S}}({\rm{TE}})={{\rm{S}}}_{0}\cdot {{\rm{e}}}^{-(\frac{{\rm{TE}}}{{\rm{T}}{2}^{\ast }})}+{\rm{C}}$$

where S_0_ represents the effective proton density, including partial T1 recovery, and the *C* term captures a rectified noise floor from magnitude imaging with possible low SNR in the final echo volume. The first echo volume of the six echo acquisition was co-registered to the fluid-nulled volume from the first imaging visit using the FMRIB software library (FSL) FLIRT tool (FSL version 4.1.9 http://fsl.fmrib.ox.ac.uk/fsl). The resulting linear transformation matrix was then applied to the T2* volume for that visit. The first echo volume was also used for automatic segmentation using the FSL FIRST tool (FSL version 4.1.9 http://fsl.fmrib.ox.ac.uk/fsl) for automated delineation of the caudate, putamen, and globus pallidus.

All six echoes were automatically summed by the custom sequence software to result in a high SNR^24^. This volume was used for the manual delineation of the substantia nigra (SN), red nucleus (RN), and the dentate nucleus (DN).

### Statistical Analysis

All statistical analyses were performed using SPSS statistical software (version 21 SPSS Inc., Chicago, Illinois) and SAS 9.3.

Demographic data were compared between PD and HV groups using independent t-tests.

The statistical methodology used in the analysis was Mixed Models applied to longitudinal data. This method takes into account the correlation between the time points (screening visit, third and sixth months) within the different brain regions of interest. Estimated values from T2* values means were analysed using Pairwise comparisons with post-hoc Bonferroni correction using T2* as dependent variable. The threshold for statistical significance was set to p < 0.05.

Correlation analysis between IL-6, TNF-alpha and ferritin with T2* regional values were performed with non parametric Spearman tests with post hoc Benjamini-Hochberg corrections for multiple correlations.

## Results

### Demographics

Demographics of enrolled patients are summarized in Table 1.

### Clinical Parameters

#### Side effects of deferiprone therapy

Deferiprone therapy was essentially well tolerated by PD subjects with only minor side effects being reported. Seven PD subjects reported an exacerbation of pre-existing muscular/joint pain, while another 3 PD patients experienced mild gastrointestinal upset during the first months of the study, all of which resolved without any clinical intervention. In one PD patient we detected an increase of liver enzymes after 4 weeks of deferiprone administration which returned to normal values after a 7-day break from deferiprone therapy. In two PD subjects administered deferiprone, a reduction in neutrophil cell numbers was observed warranting drug withdrawal. In the first subject the neutrophil decline was very rapid and severe, with zero cells being detected. In the second subject there was a slower decline in neutrophil numbers and the subject was withdrawn from the drug prior to the neutrophil numbers reaching critical levels. These subjects were monitored subsequently and their neutrophil numbers returned quickly to within normal values after deferiprone withdrawal.

#### Neurological assessment

**Figure 2:**
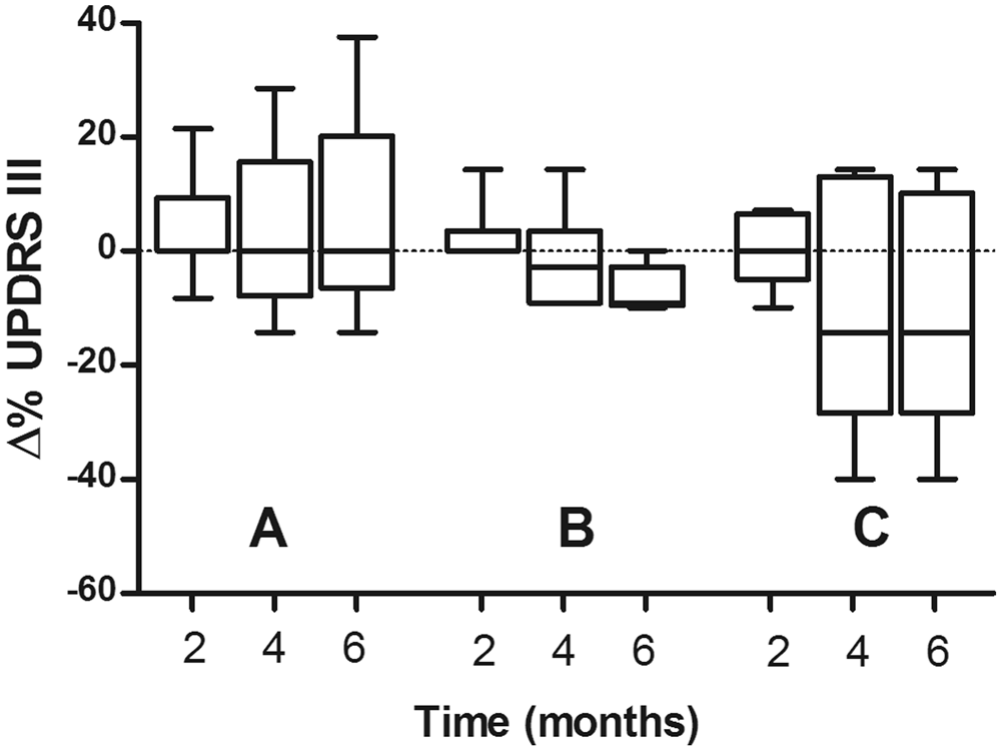
Percentage change in the median UPRDS III motor scale of PD patients receiving placebo (**A**, n = 8), 20 (**B**, n = 6) or 30 (**C**, n = 5) mg/kg/deferiprone for 2, 4 and 6 mg of treatment. Values are presented in box plots with whiskers representing the minimum and maximum values.

In the placebo-administered and 20 mg/kg/day deferiprone PD groups the motor-UPDRS scores increased over the 6 months’ treatment period which indicated a slight worsening of motor disability (Fig. 2A), however this did not reach significance. Conversely, there was a trend for a decrease in motor-UPDRS scores over the 6-month treatment period in the 30 mg/kg/day deferiprone treatment groups compared to their baseline value prior to drug treatment, indicative of improvement, but this did not reach significance (Fig. 2A). A similar but more pronounced trend was observed with the PDQ-39 scores, with the placebo and 20 mg/kg/day PD groups showing strong trends for worsening quality of life whilst the 30 mg/kg/day deferiprone PD group showed a slight improvement over 6 months but this did not reach statistical significance (Fig. 2B). There were no changes in the clinical rating obtained from the minimental State of Folstein or the Montgomery-Asberg depression scale within the placebo or deferiprone drug treated groups throughout the study (data not shown).

### MRI

**Figure 3:**
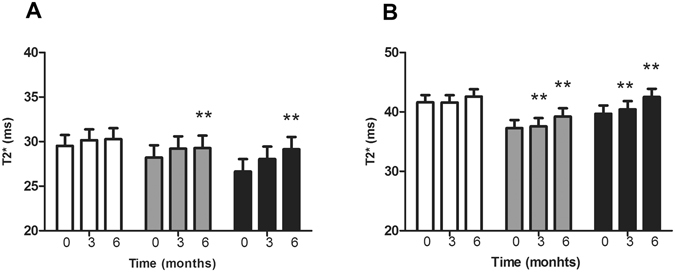
Mean T2* MRI values in the dentate nucleus (**A**) and caudate nucleus (**B**) of Parkinson’s disease subjects receiving 20 mg/kg/day, (grey bars, n = 6) or 30 mg/kg/day (black bars n = 5) mg/kg/day deferiprone or placebo (open bars, n = 8) at 3 months and 6 months. Values are represented as mean ± SEM. T2* value is prior to the commencement of treatment, and then after 3 and 6 months of drug treatment. **p < 0.01 as assessed by Pairwise comparison.

Administration of 20 or 30 mg/kg/day deferiprone resulted in a significant time-dependent decrease in iron concentrations in both the dentate and caudate nuclei, (Fig. 3). An increase in the mean T2* MRI values was evident in the dentate nucleus: the values increased significantly from 29.2 ± 0.8 to 30.6 ± 0.9 and from 27.2 ± 1.6 to 29.9 ± 1.1 (p < 0.001) after six months of 20 mg/kg and 30 mg/kg/day deferiprone administration, respectively, while no changes were evident in the placebo group (Fig. 3A). Similarly, in the caudate nucleus: T2* values increased significantly in both treatment groups from 38.9 ± 1.5 to 41.1 ± 3.4 (p = 0.007) in the 20 mg/kg/day group and from 40.8 ± 1.7 to 44.6 ± 1.6 (p = 0.0002) in the 30 mg/kg/day group after 6 months of therapy (Fig. 3B). Across each treatment group there were no significant changes in T2* mean values for SNc, red nucleus, putamen or globus pallidus in PD patients receiving either 20 mg/kg/day or 30 mg/kg/day over the period of the study (Table 2). However, we did observe a decrease in SNc iron concentration with deferiprone therapy in three patients, two of whom were hypoferritinemic by the end of the study.

**Table 2:** Mean T2* values of six brain region in PD patients analysed at 0, 3 and 6 months after administration of either 20 mg/kg/day or 30 mg/kg/day deferiprone or receiving placebo.

	Months	Substantia Nigra	Red Nucleus	Dentate	Caudate	Putamen	Pallidum
**Placebo**	0	28.09 ± 1.52	27.37 ± 0.82	30.51 ± 0.59	43.92 ± 1.22	37.26 ± 1.18	27.41 ± 1.39
	3	29.36 ± 1.84	28.76 ± 1.06	30.54 ± 0.86	43.65 ± 1.62	35.76 ± 0.90	26.25 ± 0.58
	6	28.22 ± 1.81	28.58 ± 1.23	30.74 ± 0.65	45.98 ± 2.08	36.55 ± 0.86	26.43 ± 0.87
**20** **mg**/**kg**/**day**	0	26.41 ± 1.25	28.10 ± 1.19	29.14 ± 0.84	38.86 ± 1.53	33.99 ± 1.80	25.55 ± 1.23
	3	27.05 ± 1.23	26.86 ± 1.56	30.15 ± 0.49	40.29 ± 1.74 (**)	33.54 ± 1.27	25.43 ± 1.08
	6	27.68 ± 1.06	27.78 ± 1.05	30.59 ± 0.87 (**)	41.05 ± 3.43 (**)	33.94 ± 1.75	25.89 ± 0.96
**30** **mg**/**kg**/**day**	0	23.89 ± 2.24	27.21 ± 1.56	27.16 ± 1.56	40.75 ± 1.68	33.67 ± 2.14	25.72 ± 1.76
	3	23.74 ± 1.84	26.99 ± 1.04	29.04 ± 1.82	44.69 ± 1.93 (**)	37.24 ± 2.31	24.97 ± 1.14
	6	24.57 ± 1.69	27.55 ± 1.12	29.86 ± 1.10 (**)	44.55 ± 1.57 (**)	38.19 ± 2.04	26.26 ± 1.33

Results are presented as mean ± standard error. **p < 0.01.

No significant correlations regional iron accumulation measured with T2* were found with UPDRS-III scores or disease duration.

### Blood parameters

#### Plasma levels of deferiprone and deferiprone glucuronide

PD patients who received the higher dose of the chelator, 30 mg/kg/day, showed a significantly higher concentration of both deferiprone and deferiprone glucuronide than those administered 20 mg/kg/day, confirming that the patients had conformed to the instructions of the clinical trial (data not shown).

### Haematological and biochemical parameters

**Figure 4:**
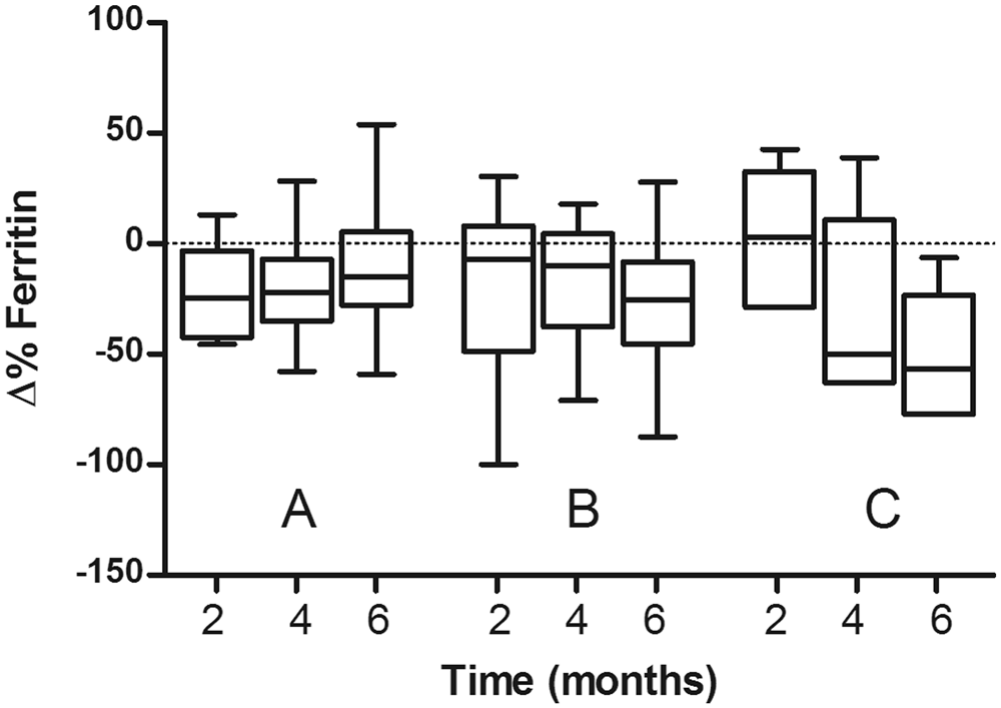
Percentage change in the median plasma ferritin values of PD patients receiving placebo (**A**, n = 8), 20 (**B**, n = 6) or 30 (**C**, n = 5) mg/kg/deferiprone for 2, 4 and 6 mg of treatment. Values are presented in box plots with whiskers representing the minimum and maximum values.

**Figure 5:**
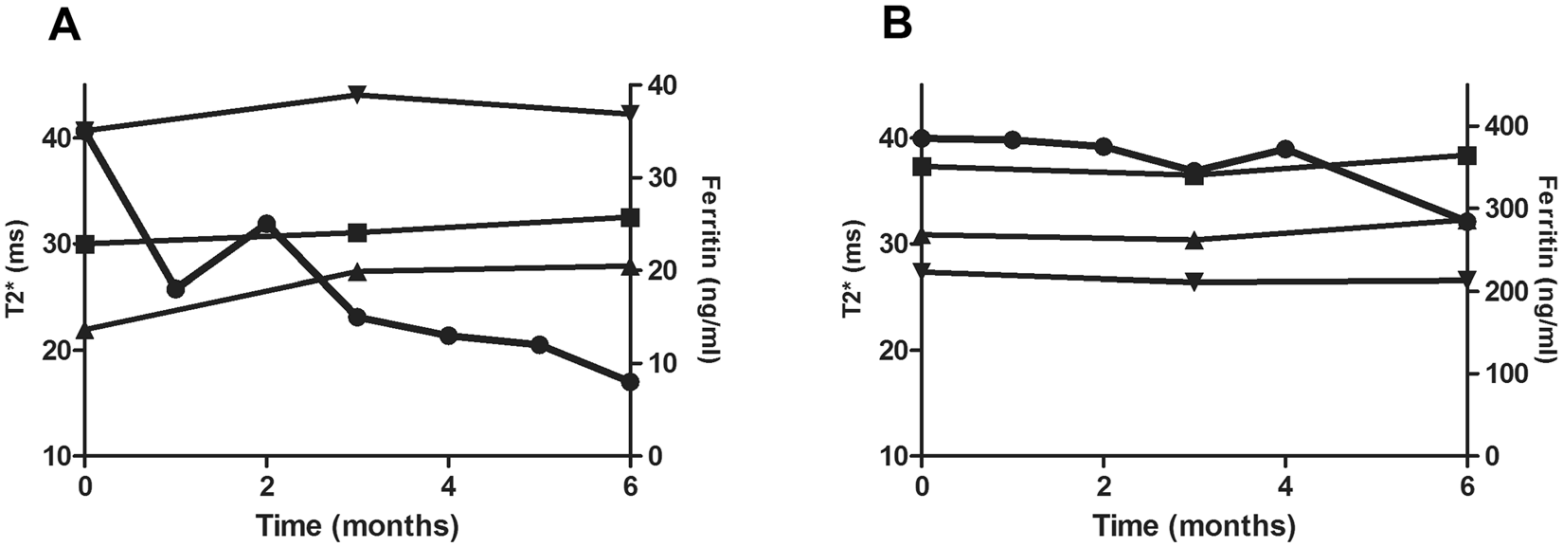
Examples of changes in ferritin levels and T2* values in two PD patients receiving chelation therapy for 6 months. (**A**) Plasma ferritin level <100 ng/ml at the beginning of the study (**B**) plasma ferritin level >300 ng/ml. Ferritin values are denoted by circles, caudate nucleus T2* values by upright triangles, dentate nucleus values by downward triangles and substantia nigra by squares.

Haemoglobin, transferrin and serum iron levels, transferrin saturation, and blood cell counts did not differ between placebo administered and deferiprone-treated patient groups during the 6-month period of the clinical study (Table 3). Ferritin concentrations in the PD patient groups administered either 20 or 30 mg/kg/day deferiprone gradually declined over the 6-month period, compared to the PD placebo group, which remained comparable to baseline values (Fig. 4). In two PD patients where the initial ferritin value was <100 ng/ml, it was noteworthy that there was a greater decrease in brain iron in the dentate and caudate nuclei, as well as the SNc, as assessed by T2* MRI, after iron chelation (Fig. 5A) when compared to other PD patients where the plasma ferritin level commenced at values >100 ng/ml (Fig. 5B).

**Table 3:** Haematological and iron status measurements expressed as medians +/− SEM of Parkinson’s disease patients receiving 20 mg/kg/day (n = 6) or 30 (n = 5) mg/kg/day deferiprone or placebo (n = 8) for 6 months.

	Months	Haemoglobin (10^9^/L)	WBC (g/L)	Neutrophils (x10^9^/L)	Platelets (x10^9^/L)	Ferritin (ng/ml)	Serum Iron (nmol/L)	Transferrin Saturation (%)
***Placebo***	**0**	141 ± 0.59	6.23 ± 0.41	3.95 ± 0.33	223 ± 18	100 ± 27	14 ± 1.2	25 ± 2.7
	**2**	137 ± 0.61	5.58 ± 0.33	3.33 ± 0.20	222 ± 15	77 ± 19	16 ± 1.4	25 ± 2.6
	**4**	137 ± 0.63	5.98 ± 0.41	3.36 ± 0.31	219 ± 15	82 ± 21	15 ± 1.8	27 ± 4.1
	**6**	137 ± 0.55	5.78 ± 0.29	3.37 ± 0.13	220 ± 12	88 ± 21	17 ± 1.4	25 ± 2.9
***20*** ***mg***/***kg***/***day Deferiprone***	**0**	136 ± 0.67	6.70 ± 0.81	4.08 ± 0.66	222 ± 20	189 ± 62	21 ± 2.1	32 ± 5.5
	**2**	132 ± 0.81	6.78 ± 0.62	4.30 ± 0.59	237 ± 22	187 ± 62	22 ± 1.9	36 ± 5.4
	**4**	134 ± 0.63	6.93 ± 0.97	4.48 ± 0.78	215 ± 21	165 ± 59	18 ± 2.2	30 ± 2.4
	**6**	135 ± 0.63	6.61 ± 0.62	4.30 ± 0.56	224 ± 23	141 ± 47	18 ± 1.1	33 ± 6.6
***30*** ***mg***/***kg***/***day Deferiprone***	**0**	141 ± 0.48	6.73 ± 0.66	4.38 ± 0.38	249 ± 17	132 ± 37	17 ± 1.9	24 ± 2.2
	**2**	134 ± 0.41	6.38 ± 0.42	4.26 ± 0.20	229 ± 14	136 ± 50	21 ± 2.5	28 ± 1.9
	**4**	140 ± 0.27	5.63 ± 0.33	3.70 ± 0.23	217 ± 14	115 ± 41	20 ± 1.9	30 ± 1.7
	**6**	138 ± 0.32	6.05 ± 0.64	4.20 ± 0.53	223 ± 17	107 ± 38	19 ± 1.2	28 ± 2.9

WBC - white blood cells.

### Urinary iron content

The iron content of spot urinary samples was assessed at monthly intervals over the 6-month trial period. There was an increase in the median value of urinary iron values in PD patients over the six month period who received deferiprone therapy, 20 mg/kg/day and 30 mg/kg/day; 172 ± 160 and 132 ± 104 nmol Fe/mmol creatinine respectively, compared to 23.1 ± 4.8 nmol Fe/mmol creatinine in the placebo group.

### Plasma Inflammatory markers

**Figure 6:**
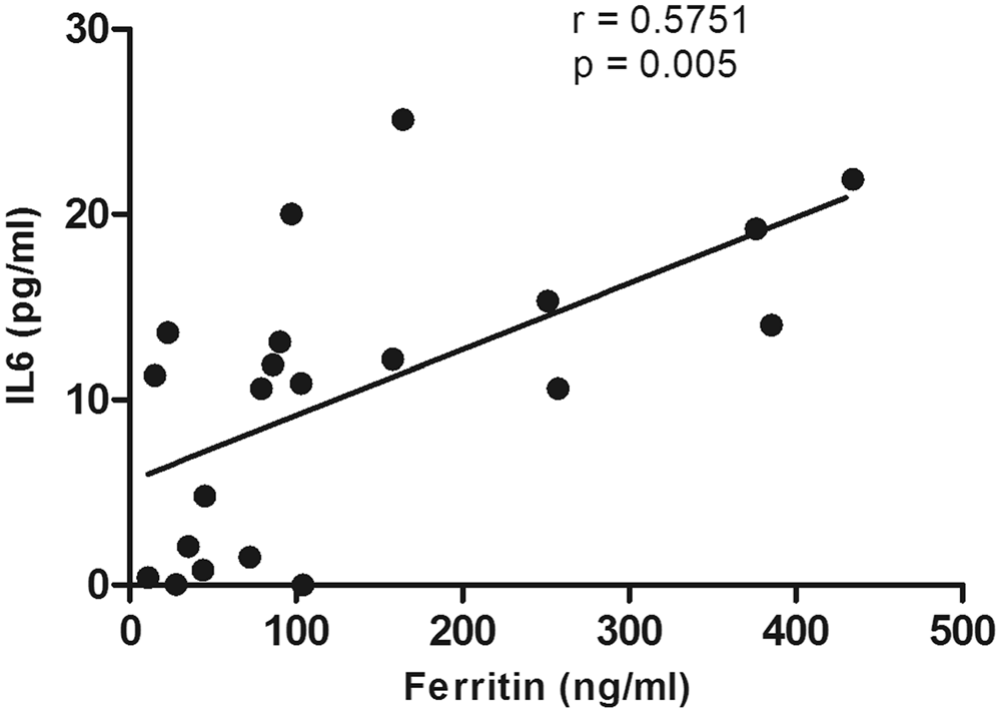
Correlation between IL-6 (pg/ml) and ferritin (ng/ml) baseline values from all clinical trial PD subjects. Statistical evaluation is by non-parametric correlation of Spearman (r = 0.5751, p = 0.005).

Plasma concentrations of IL-6 and TNFα were analysed at monthly intervals during the 6-month clinical trial period. Whilst there were no changes in IL-6 and TNFα concentration with time in either the placebo or deferiprone treated PD groups, there was a positive correlation between the plasma levels of IL-6 and ferritin at Time 0 (Fig. 6). No further correlations were found between IL-6, TNF alpha, ferritin and regional T2* values at baseline and follow up visits.

## Discussion

This pilot clinical trial with the iron chelator deferiprone clearly demonstrated that over the 6 months’ treatment period there was gradual iron removal from specific brain regions, namely the caudate nucleus and dentate nucleus, as assessed by T2* MRI, in the majority of the PD patients while only three subjects showed alterations in the T2* MRI values for SNc. Deferiprone therapy was well tolerated by the PD patients, with a few patients experiencing mild side effects, while a decline in white cell counts warranting drug withdrawal was evident in 2 PD subjects. Deferiprone administration did not adversely affect PD severity, mood, cognitive function or quality of life or peripheral iron status. Furthermore, serum ferritin levels appear to be a surrogate marker to identify which patients might respond optimally to iron chelation by deferiprone. Additionally, plasma ferritin levels correlated with IL-6 concentrations, implicating a link between peripheral inflammation and iron metabolism in PD.

Our MRI findings of a decrease in the iron content of the dentate nucleus correlate with the clinical trial in FA patients carried by Boddaert and colleagues^18^, who also utilised 20 or 30 mg/kg/day doses of deferiprone over 6 months. Furthermore, Velasco-Sanchez *et al*.^25^ administered 20 mg/kg/day deferiprone in addition to the antioxidant idebenone, to FA patients and showed an increase in T2* values in dentate nucleus after 11 months. Nevertheless we did not find any association between tremor sub-scores and dentate iron accumulation.

In this present study, short-term deferiprone treatment was only observed to remove iron from the SNc in three patients receiving deferiprone. This could possibly be explained by the fact that iron in the SNc is principally tightly bound to neuromelanin rather than to ferritin as in other brain regions and hence less chelateable in the SNc^26, 27^. Hence, a longer period of deferiprone therapy may be required before SNc iron levels decline. However, importantly this study demonstrates that deferiprone therapy does not result in a generalised removal of iron from the brain or its redistribution which could have implications for other neurological functions. This was supported by the finding that 6 months deferiprone therapy did not adversely affect mood, cognitive function or general patient quality of life. However, some of these data differ from another clinical trial of deferiprone therapy in PD patients, the FAIRPARK clinical trial, where deferiprone at 30 mg/kg/day was administered to PD subjects either at the initiation of the trial (‘early start subjects’), or after 6 months of placebo therapy (‘delayed start group’)^28^. Both ‘early start’ and ‘delayed start’ PD subjects showed a small but significant reduction in SNc iron after 6 months of therapy as assessed by MRI^28^. This small effect may have achieved significance possibly due to the increased cohort size. Additionally, only one dose of deferiprone, 30 mg/kg/day, was used in the 19 patients participating in the FAIRPARK study, compared to 11 subjects receiving either 20 or 30 mg/kg/day in this study, thus giving the FAIRPARK study greater power to detect significant small changes in MRI signal.

A further difference between the FAIRPARK study and this study was that the former observed a small but significant clinical improvement in the deferiprone-treated PD subjects compared to placebo-treated PD subjects^28^. The delayed start PD subjects who received placebo over the first 6 months averaged a 1 point UPDRS decline in motor function, whilst the early start deferiprone group improved by an average of 2 UPDRS points over the same period, giving an overall UPDRS difference between the two groups of 3 points. In our pilot study we observed a trend for an improvement in UPDRS motor scores in the 30 mg/kg/day deferiprone treated subjects compared to the placebo and 20 mg/kg/day group although this was not significant. This may again relate to the FAIRPARK study utilising the more active dose of 30 mg/kg/day dose of deferiprone for the whole of their study and in a slightly greater number of subjects. In addition in the FAIRPARK study, PD patients (n = 19) who were recruited, had a clinical diagnosis of PD made less than 3 years before enrolment, whereas subjects recruited for this present study had a disease duration of less than 5 years. However the FAIRPARK patients showed higher motor scores UPDRS = 23.7 ± 7 by comparison to the PD patients in the present study, 11 ± 0.8, the former having been assessed in off medication state.

As in the FAIRPARK study we also observed a reduction in serum ferritin concentrations in PD subjects treated with deferiprone compared to placebo. This would reflect the mobilisation of iron stores particularly from the liver. With regards to brain iron mobilisation, deferiprone therapy was observed to be most efficient in those PD subjects starting with low serum ferritin content <100 ng/ml at the start of the study. This might suggest that there is a need to lower body iron stores before iron can be mobilised from specific brain regions. Indeed, in animal studies where body iron stores were depleted, there was an increased release of iron from the brain^29^. There was a correlation between the plasma concentrations of ferritin and IL-6, suggesting that the iron status of PD patients with high plasma ferritin may reflect the anaemia of chronic disease. Indeed, increased levels of pro-inflammatory cytokines, IL-1β, IL-2, IL-6 and TNFα are present in the brain, cerebrospinal fluid^30^ and plasma of PD patients^31–33^ such that changes in IL-6 observed in PD plasma might be indicative of inflammatory changes in the brain^34^. It is unknown whether plasma hepcidin levels, the master regulator of iron metabolism, are altered in PD patients. Hepcidin binds to the sole known iron exporter, ferroportin, causing its internalisation and degradation, thereby inhibiting iron efflux^35^. Hepcidin transcription can be stimulated by increases in serum iron as well as by inflammatory stimuli, most notably by IL-6, which signals via the IL6 receptor (IL-6Ra) and gp130 via the janus kinase (JAK) – STAT 3 pathway^36, 37^; such pathways are important since they can lead to the internalisation of iron in cells.

In conclusion, we have demonstrated that short term deferiprone therapy in PD subjects is safe and is associated with decreases of iron in specific brain regions. These studies support future longer-term clinical trials in PD where the neuroprotective effects of deferiprone can be fully assessed.
